# Statistical methods and graphical displays of quality of life with survival outcomes in oncology clinical trials for supporting the estimand framework

**DOI:** 10.1186/s12874-022-01735-1

**Published:** 2022-10-04

**Authors:** Kentaro Sakamaki, Takuya Kawahara

**Affiliations:** 1 Center for Data Science, Yokohama City University, 22-2 Seto, Kanazawa-ku, Yokohama, 236-0027 Japan; 2 Clinical Research Promotion Center, The University of Tokyo Hospital, Tokyo, Japan

**Keywords:** Quality of life, Truncation by death, Estimand framework, Graphical displays, Prioritized composite outcome, Semi-competing risk analysis, Principal stratification

## Abstract

**Background:**

Although there are discussions regarding standards of the analysis of patient-reported outcomes and quality of life (QOL) in oncology clinical trials, that of QOL with death events is not within their scope. For example, ignoring death can lead to bias in the QOL analysis for patients with moderate or high mortality rates in the palliative care setting. This is discussed in the estimand framework but is controversial. Information loss by summary measures under the estimand framework may make it challenging for clinicians to interpret the QOL analysis results. This study illustrated the use of graphical displays in the framework. They can be helpful for discussions between clinicians and statisticians and decision-making by stakeholders.

**Methods:**

We reviewed the time-to-deterioration analysis, prioritized composite outcome approach, semi-competing risk analysis, survivor analysis, linear mixed model for repeated measures, and principal stratification approach. We summarized attributes of estimands and graphs in the statistical analysis and evaluated them in various hypothetical randomized controlled trials.

**Results:**

Graphs for each analysis method provide different information and impressions. In the time-to-deterioration analysis, it was not easy to interpret the difference in the curves as an effect on QOL. The prioritized composite outcome approach provided new insights for QOL considering death by defining better conditions based on the distinction of OS and QOL. The semi-competing risk analysis provided different insights compared with the time-to-deterioration analysis and prioritized composite outcome approach. Due to the missing assumption, graphs by the linear mixed model for repeated measures should be carefully interpreted, even for descriptive purposes. The principal stratification approach provided pure comparison, but the interpretation was difficult because the target population was unknown.

**Conclusions:**

Graphical displays can capture different aspects of treatment effects that should be described in the estimand framework.

**Supplementary Information:**

The online version contains supplementary material available at 10.1186/s12874-022-01735-1.

## Introduction

In oncology clinical trials, quality of life (QOL) and patient-reported outcomes (PROs) are incorporated to evaluate the benefits and risks of cancer treatments, along with survival outcomes, such as overall survival (OS) or progression-free survival. For instance, the Food and Drug Administration guidance [1] considers treatment effects on OS as fundamental for the approval of cancer drugs, and PROs can be used to support labeling claims [2]. They can facilitate regulatory, patients’, and clinicians’ decision-making [3–5].

The Setting International Standards for the Analysis of Quality of Life (SISAQOL) Consortium proposed standards for the statistical analysis of QOL and PROs [6]. They recommended two approaches: the Cox proportional hazards for time to improvement/(end of) stable state/worsening and linear mixed models for repeated measures (MMRM) for the magnitude of improvement or worsening at measured time points and response patterns or profiles [6]. These approaches can be easily applied if all PROs are measured without death and truncation. The standards [6] mention that “PRO assessments after death should not be expected because a meaningful value for these observations will not exist” and that “these assessments are also not meaningful for analysis because they will not have a relevant contribution to the PRO estimate.” ICH E9 (R1) [7] also mentioned that “for terminal events such as death, the variable cannot be measured after the intercurrent event, but neither should these data generally be regarded as missing.” However, in oncology clinical trials for patients with high mortality rates, for instance, palliative care or immunotherapy clinical trials [8, 9], PROs are important and cannot be evaluated without considering death. These issues are discussed in the estimand framework [10], and it is worthwhile to review statistical methods that account for death (survival outcomes) in the estimand framework.

Regarding statistical methods used to compare PROs in randomized controlled trials with high mortality, Colantuoni et al. [11] illustrated the survivor analysis, survivor average causal effect (SACE) [12, 13], and composite endpoint approaches. They are related to strategies for addressing death as intercurrent events in the estimand framework; the hypothetical, composite variable, while on treatment, and principal stratum strategies [7]. In PROs analysis considering survival outcomes, the MMRM and time-to-event (TTE) approaches can be applied under the hypothetical and composite variable strategies, respectively. However, these methods have some deficiencies related to the assumption of missing mechanisms [14], priority of events types [15], and assumptions embedded in the analytical approaches and definitions of endpoints [16]. For example, the MMRM might need missing at random assumption, and the TTE approach does not consider the priority of death and worsening PROs. Hence, the prioritized composite outcome approach [11, 17, 18], terminal decline conditional analysis [19, 20], and semi-competing risks analysis [21] can be alternative and informative methods.

The analysis results of PROs using these statistical methods can be complicated for non-statisticians (including clinicians and patients) to understand due to some reasons. First, consideration of the estimand framework in the PROs analysis is uncommon [10] or new [22]. Second, there are many effect measures (or summary measures) for each statistical method; the hazard ratio, survival probabilities, and restricted mean survival times [23] in TTE analysis. Ease of interpretation is desirable to guide informative decision-making and to influence clinical practice [6]. Interpretation can be facilitated using graphical displays to make complex information visually salient [2, 24, 25]. Statistical results should be complemented by graphical displays [6]. However, to the best of our knowledge, there is no discussion of statistical methods and graphical displays regarding analysis of PROs considering survival outcomes to facilitate the interpretation of treatment effects in the estimand framework.

This article discusses key aspects and graphical displays in statistical methods for QOL (or any other PROs) analysis considering survival outcomes in randomized clinical trials. Specifically, we outline the attributes of estimands (population, variables, strategies for dealing with death, and effect measures) and graphical displays in the statistical methods. We applied these methods for evaluating composite variables and QOL itself in hypothetical randomized clinical trials. We introduce some effect measures and focus on the explanation of graphs that support the interpretation of these measures.

## Methods

### Statistical methods and graphical display

There are methods for summarizing QOL and those for estimating treatment effect using composite variables or QOL itself. The methods for evaluating composite variables include time-to-deterioration (TTD) analysis, the prioritized composite outcome approach, and semi-competing risk analysis. Those for QOL itself include the survivor analysis approach, the terminal decline approach, the MMRM, and principal stratification for SACE. The survivor analysis [11] and terminal decline approaches [26] are used for summarizing rather than estimating the treatment effect on the QOL itself (often calculates the mean and standard deviation). In summarization methods, the survivor analysis summarizes QOL data restricted to those of survivors at a specific time point [11], the terminal decline approach also summarizes restricted QOL data based on the time scale that counts backward from death [26].

We consider a randomized controlled trial comparing new and standard treatments and outline the attributes of estimands, strategies for dealing with death, and graphical display in Table 1 for statistical methods other than summarization methods.

**Table 1:** Attributes of estimands and graphical display of statistical methods

Objective	Attributes				Graphical display
	Population	Variables (endpoints)	Strategies for dealing with death	Effect measures	
**Time-to-deterioration (TTD) analysis (method for evaluating composite variables)**					
• Evaluate time to deterioration (death or deterioration)	• All participants	• Time to first event (death or deterioration)	• Composite endpoint • Death and deterioration are equally treated	• Event probability at a specified time • Hazard ratio • Median (or mean) time to event	• Describe the proportion of deterioration by survival curves
**Prioritized composite outcome approach (method for evaluating composite variables)**					
• Evaluate “win” by multiple aspects (multiple endpoints)	• All participants	• Composite endpoint by death and deterioration • “Win” defined by generalized pairwise comparisons	• Composite endpoint • Requires eliciting expert opinion on ordering death and QOL	• Win ratio [27] • Net benefit [15]	• Describe the combination of two step charts of cumulative probability of death and that of QOL deterioration
**Semi-competing risk analysis (method for evaluating composite variables)**					
• Evaluate the cumulative probability of QOL deterioration having occurred in the presence of death	• All participants	• Time to deterioration	• Competing risks	• Sub-distributional hazard ratio • Cause specific hazard ratio	• Describe the cumulative probability of QOL deterioration (cumulative incidence function)
**Linear mixed model for repeated measures (MMRM) (method for evaluating QOL itself)**					
• Evaluate the magnitude of worsening QOL	• All participants	• QOL at the time of primary interest • QOL at every visit	• Assuming missing at random for death • Implicitly impute data beyond death	• Mean difference in QOL at the time of primary interest • Difference in slopes of QOL trajectories over time	• Describe a trajectory of mean QOL over time using a line chart with a measure of uncertainty
**Principal stratification for survivor average causal effect (SACE) (method for evaluating QOL itself)**					
• Evaluate the magnitude of worsening QOL	• Participants who would not die regardless of which treatment they received (“always survivors”)	• QOL at the time of primary interest • QOL at every visit	• Death in the target population does not occur • The target population is not directly identifiable	• Mean difference in QOL at the time of primary interest • Difference in slopes of QOL trajectories over time	• Describe a trajectory of QOL over time by line chart with a measure of uncertainty

*QOL* quality of life

The following sections explain the prioritized composite outcome approach, semi-competing risk analysis, and principal stratification for SACE, because the MMRM and TTD analysis were previously described in-depth and tailored for non-statisticians [28]. It should be noted that the graphs for the MMRM and TTD analysis are trajectories of average QOL and the Kaplan-Meier curves for survival outcomes.

### Prioritized composite outcome approach

In this approach, a variable (endpoint) is defined as a composite endpoint considering the priority (ordering) of multiple outcomes, such as survival outcome and QOL. According to the study objectives and clinical aspects, the definition of the prioritized composite outcome can vary [17, 18, 27, 29]. Based on Lachin [17] and Colantuoni [11], this study considered the priorities from worst to best as follows: 1) time to death (earlier death is considered worse) and 2) QOL among survivors at a specific time point *t* (lower QOL is considered worse among survivors). To define a prioritized composite outcome that higher scores correspond to a better condition, we considered a score *U* as follows:

$$U=\left\{\begin{array}{c}\mathrm{survival}\ \mathrm{time}\ \mathrm{if}\ \mathrm{death}\ \mathrm{until}\ \mathrm{time}\ t\\ {}\mathrm{QOL}+t\ \mathrm{if}\ \mathrm{survival}\ \mathrm{at}\ \mathrm{time}\ t\end{array}\right.$$

Depending on specifying time *t*, censoring, truncation of QOL, and intercurrent events other than death, it may be necessary to change the definition of the prioritized composite outcome. For example, *t* = 12 months or 5 years in consideration of the clinical context.

As *U* can be defined for all participants in a clinical trial, we can evaluate the treatment effect under the intent-to-treat principle [7]. When defining effect measures, the mean (expected value) of *U* cannot be used because *U* is defined based on two components with different scales. Statistical inferences based on ranks can be used, such as percentile (median) or the Mann-Whitney-Wilcoxon test [11]. Therefore, the effect measure related to the estimand should be carefully defined when using the prioritized composite outcome.

To clarify the two components with different scales in graphs, a graph was used for the prioritized composite outcome as Colantuoni et al. [11] and Wang et al. mentioned [30]: 1) cumulative incidence curve of survival time until time *t* and 2) cumulative distribution of QOL among survivors at time *t*. The scale of the x-axis in the graph is time until time *t* and then QOL at time *t*. That is, the cumulative incidence curve after time *t* is replaced with a cumulative distribution of QOL. In practice, scales (or widths) of time and QOL make different impressions. Some illustrative graphs are shown in the below section.

### Semi-competing risk analysis

In the semi-competing risk analysis [21, 31], we can focus on the deterioration (worsening) of QOL as an event of interest and death as a competing event. Unlike other composite endpoint approaches, such as the TTD analysis (time to first event in deterioration of QOL and death) and prioritized composite outcome, the events of different types are evaluated separately. Therefore, the semi-competing risk analysis should be considered useful for evaluating components of composite endpoints [32]. Notably, deaths after a deterioration of QOL may be observed, although the reverse would not be observed.

Time-to-deterioration of QOL is a variable in the estimand framework. As we can define it among survivors and those who died without deterioration of QOL (i.e., time-to-deterioration is “postponed” to infinity), treatment effects can be evaluated under the intent-to-treat principle. Time-to-deterioration of QOL can be summarized using a cumulative incidence function (crude risk function), which is related to a sub-distribution hazard function [33, 34]. Hence, we can use Gray’s test [35] and the Fine–Gray model [36] for the comparison of treatment groups.

In the semi-competing risk analysis, the cumulative incidence function is graphically presented. As it is difficult to interpret the function without information of competing events [33, 34], a graphical display of the survival function should be given. For example, when the OS event always precedes the QOL event, the absence of the QOL event cannot be interpreted as reducing it due to the treatment. Therefore, it is crucial to interpret the graph along with the Kaplan–Meier curve of OS.

### Principal stratification for survivor average causal effect

Whereas the composite variable strategies consider death and QOL for the definition of the variable, when examining the treatment effect on QOL, QOL itself should be a variable in the estimand framework. If the treatment has a causal effect on survival, survivor analysis yields biased estimates of causal effects on QOL even in randomized trials [37]. If the QOL value of “patients who died” can be reasonably defined, for instance, by assigning 0 for death, it can be analyzed causally under the intent-to-treat principle. However, few questionnaires explicitly define QOL corresponding to death [38]. In situations where QOL after death cannot be defined, the SACE under the principal stratification framework can define useful causal effects [11–13].

For the SACE, the population in the estimand framework comprises participants who would survive until the time of QOL measurement regardless of treatment. Those participants are called “always survivors,” and the SACE is the treatment effect among them [13]. Its advantage over survivor analysis is that it avoids the problem of selection bias due to conditioning on living participants [13]. However, it is not possible to know which participants belong to the “always survivor” group because their outcomes are observable under only one of the treatments.

To estimate the SACE at each time point, it is often necessary to assume an explainable non-random survival assumption [39]. This assumption implies that QOL with one treatment does not further predict survival with another treatment given baseline covariates. Therefore, survival with treatment can be predicted by baseline covariates. Based on this key assumption, the contrast between treatment groups about observed QOL weighted by the predicted survival probability provides a consistent estimate for the SACE [40]. Testing about the SACE, within pre-exposure covariate levels, has been proposed [41].

In this analysis, SACE components (i.e., an average of each treatment group) at each time point can be plotted the same as in the MMRM or survivor analysis. However, it should be recognized that the analysis population comprises “always survivors” and differed from the MMRM or survivor analysis.

### Illustrative examples of hypothetical randomized controlled trials

We compared graphical displays between the methods for composite variables and for QOL itself in hypothetical randomized controlled trials. Four scenarios were considered and are described below.

- Scenario 1: OS is longer, and QOL is higher in the new versus the standard treatment arm.
- Scenario 2: QOL is higher, although OS is shorter in the new versus the standard treatment arm.
- Scenario 3: QOL is higher, and OS is equivalent in the new versus the standard treatment arm.
- Scenario 4: OS is longer, and QOL is equivalent in the new versus the standard treatment arm.

We did not consider a scenario wherein QOL is lower, although OS is longer in the new versus the standard treatment arm because this is equivalent to Scenario 2 with the treatment indicators flipped.

**Fig. 1:**
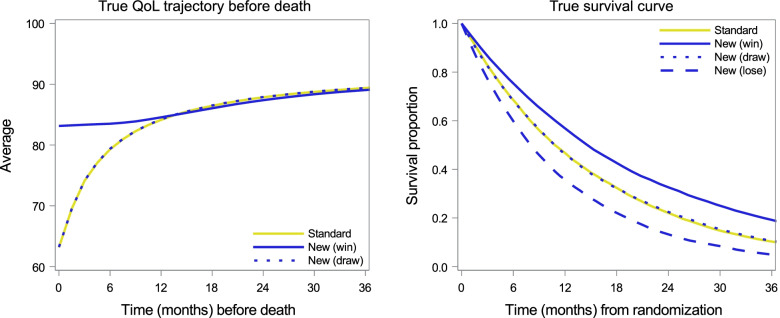
Graphs for simulated data. Left: terminal trajectories of QOL; Right: survival curves

Figure 1 summarizes data in hypothetical randomized controlled trials; survival curves of OS using the Kaplan-Meier methods; and terminal trajectories of QOL using the terminal decline approach. Trajectories of QOL when the new treatment was effective on QOL (blue and solid yellow lines) were similar to the findings of a real randomized clinical trial of palliative care [20, 42]. When there was no treatment effect on QOL, terminal declines were the same (blue dotted line and solid yellow line). For OS curves, three patterns were considered for the new treatment: hazard ratios of 0.74 (solid line), 1 (dotted line), and 1.35 (dashed line). Graphs corresponding to each scenario are shown in Supplementary Fig. 1.

In each scenario, we only generated one hypothetical trial with 20,000 patients, who were randomly assigned to receive either the new or standard treatment. This makes important aspects of graphs clear. Among these, approximately 50% of patients were independently censored. QOL was measured every 3 months. Protocol for data generation is available in Supplementary Method 1.

We applied five statistical methods described in Table 1 and the survivor analysis and obtained the graphical results. Details of methods for composite variables are as follows: The prioritized composite outcome approach focused on the time to death until 12 months, predefined survival endpoint in the trial [42], and QOL at 12 months among survivors. In the TTD and semi-competing risk analyses, a 10-point decline from baseline was defined as a QOL deterioration. Death or deterioration, whichever occurred first, was treated as an event in the TTD analysis. Details of methods for QOL itself are as follows: The QOL data obtained every 3 months were analyzed in the survivor analysis and SACE. In the MMRM analysis, all the QOL data of the observation period were used, and baseline covariates (baseline QOL and sex) were included in the exploratory variables. To highlight the conceptual difference between the SACE and other two methods, true values of SACE were generated from the simulation model, rather than estimated.

We conducted all statistical analyses for the simulation study using Base SAS and SAS/STAT version 9.4 software of the SAS System for Windows (SAS Institute, Cary, NC, USA). The program for data generation is available in Supplementary Method 2.

## Results

### Graphical results in illustrative examples

**Fig. 2:**
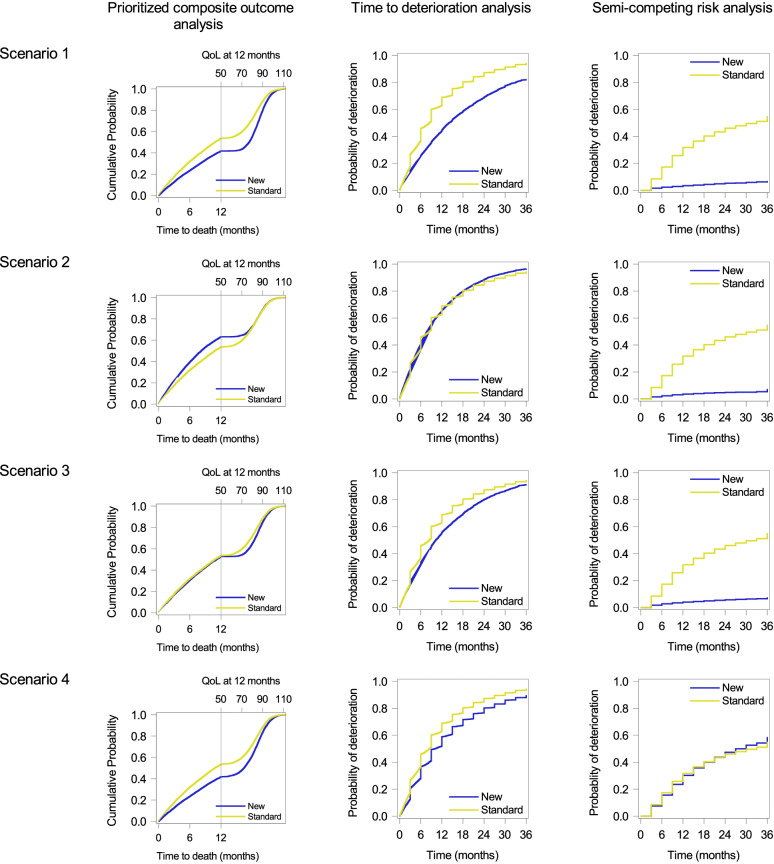
Graphical results of methods for composite variables. Top to bottom: Scenario 1 to Scenario 4. Left to right: Prioritized composite outcome analysis, time-to-deterioration analysis, and semi-competing risk analysis

Figure 2 shows graphs from methods for composite variables (left: prioritized composite outcome analysis; center: TTD analysis; right: semi-competing risk analysis) for each scenario (Supplementary Fig. 1).

In graphs by the prioritized composite outcome analysis (Fig. 2, left) evaluating a combination of time to death and QOL, the lower curve indicates better treatment; the new treatment was better in Scenarios 1, 3, and 4, and the standard treatment was better in Scenario 2. When evaluating quantiles, for example, 50 (median) and 70 percentiles of the prioritized composite endpoint in the standard and new treatments were 10.8 and 8.2 months, as well as 78.9 and 78.0 of QOL with survival, respectively. As participants who had lived at 12 months did not die until 18 months and terminal decline of QOL started 6 months before death, the graphs were similar between Scenarios 1 and 4. In Scenario 2, the graph did not show better QOL in the new treatment. For example, the 50 percentile in the standard treatment was better than that in the new treatment, whereas 70 percentiles or greater would be similar between the new and standard treatment groups. In Scenario 3, OS was comparable between the two groups and the difference in QOL well represented that the new treatment was slightly better because the terminal decline of QOL before death was large.

In graphs by the TTD analysis (Fig. 2, middle) without distinguishing OS and QOL events, the lower curve indicates better treatment; the new treatment was better in Scenarios 1, 3, and 4, and both treatments were similar in Scenario 2. As QOL events occurred every 3 months, stepped curves were observed when QOL events occurred earlier than OS events. On the other hand, smooth curves were observed when OS events occurred without QOL events. That is, the graph in Scenario 2 shows that curves were overlapped but observed events were different. In Scenario 4, the difference in the curves appears to be derived from QOL events but was derived from the treatment effect on OS. This can be known because this is a simulation study, and the data generating process was known. But in practice, it can be challenging to separate the information for QOL and OS from the graphs in the TTD analysis.

The graphs for the semi-competing risk analysis (Fig. 2, right) focus on QOL deterioration exclusively while considering OS. Due to exclusion of death as an event or censoring, these graphs provide different information and impressions from those of the TTD analysis. For example, in Scenarios 2 and 4, the difference in curves between treatment groups varied between the semi-competing risk and TTD analyses. In Scenarios 1 and 4, as the graphs by the prioritized composite outcome analysis showed QOL among survivors at 12 months, those by the semi-competing risk analysis could complement information at and after 12 months.

**Fig. 3:**
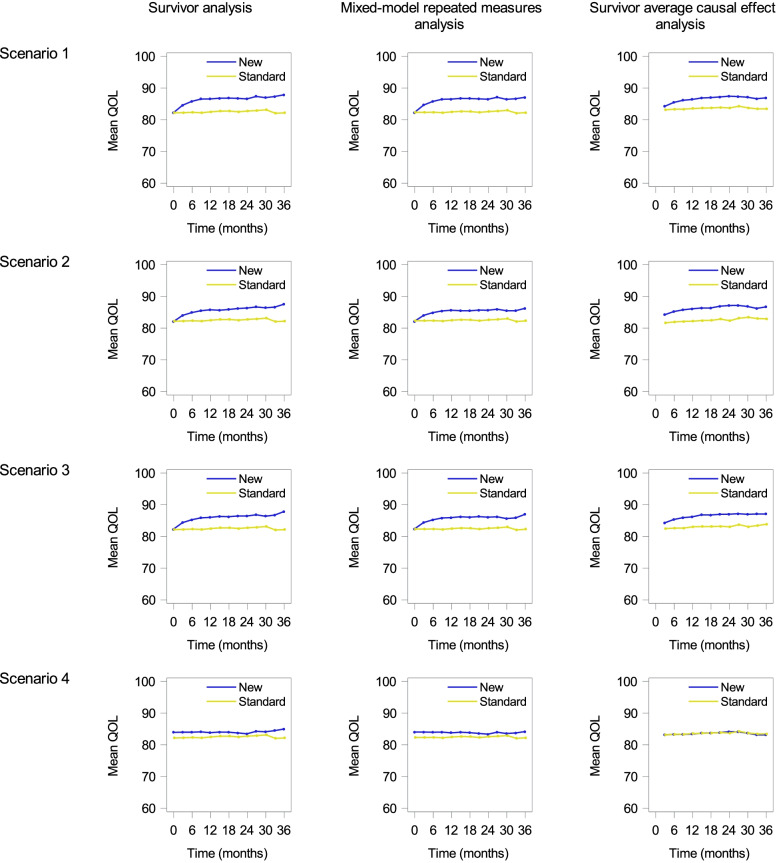
Graphical results of methods for QOL itself. Top to bottom: Scenario 1 to Scenario 4. Left to right: The survivor analysis, linear mixed models for repeated measures analysis, and survivor average causal effect analysis

Figure 3 shows the graphs from methods for QOL itself (left: survival analysis; center: MMRM; right: SACE) for each scenario (Supplementary Fig. 1). In all the graphs, upper trajectories indicate better treatment.

The graphs by the survivor analysis (Fig. 3, left) and MMRM (Fig. 3, middle) showed similar results in this example; the new treatment maintained QOL in all time-points in Scenarios 1 to 3. Although QOL dropped nearly 20 points before death and 50% of participants died at 12 months in the standard treatment group (Fig. 1), the observed differences were small. In Scenario 4, although there was no difference in terminal QOL declines between treatment groups, a new treatment that prolonged OS resulted in slightly higher QOL at each time point. These graphs may lead to the false conclusion that QOL is slightly better maintained by the new treatment. Notably, the graph by the MMRM explains that truncated QOL data due to death are not implicitly imputed [26].

The graphs for the SACE (Fig. 3, right) indicate average QOL among “always survivors.” In Scenarios 1 to 3, the graphs for the SACE were similar to those by the survivor analysis and MMRM. The difference between these two methods is evident in Scenario 4; the SACE correctly presented the no-treatment effect on QOL, while the others do not.

## Discussion

In this study, we reviewed the analysis methods for QOL considering survival outcomes in oncology clinical trials and graphical displays of the analysis results. In hypothetical randomized controlled trials, we showed differences in the characteristics of graphs and provided interpretations. Although the analysis method should be selected based on the trial objectives or the estimand framework [7, 22], it is not easy to interpret summary measures or effect measures because the attributes of the estimand can be complex and summary measures may not be representative of the distribution of variables. As graphs can provide important information for clinicians and patients to interpret the analysis results and to make treatment choices, examination of the characteristics of graphical displays is needed. Therefore, we would like researchers to choose statistical methods carefully corresponding to the trial objective in consideration of Table 1 and discussions as follows.

The MMRM and TTD analysis are frequently used in oncology clinical trials [43] and are recommended by the SISAQOL when the cause of the missing data is not death [6]. As shown in our illustrative examples, the results and interpretation of the graphs by the MMRM are not apparent when QOL data is truncated due to death. It is well known that the MMRM is invalid when the missing at random assumption is not satisfied. As QOL truncated due to death is undefined, the usual missing at random assumption does not fit this situation. Even for descriptive purposes, the MMRM could not be interpretable. In the TTD analysis, the shape of the curve and impression of the graph differ depending on whether death or QOL deterioration occurs first. This is related to which treatment effects on QOL and OS affect the curve and whether the difference between treatment groups may be eliminated or increased. For instance, in Scenario 4, the shape of the curve appears to be derived from QOL events, but the difference in the curves was derived from the treatment effect on OS. As it is not clear how the effect on OS appears on the graph, it is not easy to interpret the difference in the curves as an effect on QOL. Notably, the definition of time-to-event itself varies among studies [16, 44].

Among the graphs by methods for composite variables, the prioritized composite outcome approach provides new insights for QOL considering death. In this approach, a better condition is defined from multiple outcomes considering the clinical priority, and the graphs illustrate better conditions by scores and their quantiles. Therefore, treatment effects can be evaluated at each quantile that has different meanings. For example, in Scenario 2, the 50 percentile of the prioritized composite endpoint in the standard treatment was better than that in the new treatment, whereas 70 percentiles would be similar between the new and standard treatment groups. This major feature is not found in other analysis methods. In the graphs, the part of OS (outcome with the first priority) can be easily interpreted because it is not affected by QOL. When interpreting the part of QOL (outcome with the second priority), we see it as the proportion of survivors with better QOL. If this approach becomes more common, then this interpretation is easier than that of Kaplan-Meier curves by the TTD analysis. That is because the prioritized composite endpoint approach defines better conditions based on the distinction of OS and QOL, whereas the TTD analysis does not distinguish between OS and QOL events. There are two cautions: the definition of the prioritized composite outcome and widths of the left and right parts of the graph. For example, time *t* for the prioritized composite outcome can alter the graphs. Although we divided the left and right parts in half, the appearance and impression may change depending on how their widths are decided.

The semi-competing risk analysis gives different insights compared with the other methods for composite variables [32]. The graphs that represent the cumulative incidence of QOL events provide supporting information for the TTD analysis, along with Kaplan-Meier curves for OS. Specifically, it was important to confirm three graphs in Scenarios 2 and 4. The expression of treatment effects in the graphs of the semi-competing risk analysis differed from that in those of the MMRM. In Scenario 4, although the trajectories of QOL in the MMRM were different between treatment groups, the semi-compering risk analysis provided no difference. While it may depend on the magnitude of the treatment effect and definition of deterioration, the semi-competing risk analysis is useful. In Scenarios 1 to 3, the graphs were similar, but it may not be generalized that the treatment effect on OS does not affect the cumulative incidence of QOL events.

By the principal stratification for the SACE, QOL was fairly compared in Scenarios 1 to 4 and similar trajectories between treatment groups were shown in Scenario 4. This feature is the main advantage of the SACE. However, the analysis population (always survivors) is different from the whole participants. In Scenarios 1 to 3, graphs were similar among the methods for QOL itself, although their analysis populations were different. Survivor analysis is consistent with the SACE only when treatment does not affect OS, because all patients or survivors at some time point after randomization are comparable between groups. That is, care should be taken in the use of survivor analysis for treatment-comparison purposes because the assumption is not feasible and may have selection bias. Notably, “always survivors” at each time point were different. Therefore, these graphs should be interpreted carefully. A major disadvantage is that “always survivors” cannot be empirically known. This may be a reason why the SACE is uncommon.

This study has some limitations. First, we did not include the pattern-mixture model [26], for example, which stratifies patients who die within 3 months, die within 3 to 6 months, and survive after 6 months, describing the transition of QOL within each stratum. The interpretation of the results of the pattern-mixture model is difficult when the new treatment is effective on OS because stratification depends on treatment effects. Second, we focused on graphical display rather than effect measures. Effect measures differ depending on the aspect of treatment and disease, and the distribution of endpoints can be complicated. Therefore, we did not focus on effect measures and their bias and efficiency of estimates. It should be noted that effect measures among statistical methods cannot be compared because of “apples and oranges.” Finally, the scenarios and data generation for simulation are limited. We considered that the simulation studies successfully captured the characteristics of the graphs because the generation of QOL was the same as that of the actual randomized controlled trial of palliative care in advanced cancer [42], and some scenarios for OS were considered. However, notably, not all oncology clinical trials yield the same graphical pattern as that in Figs. 2 and 3.

## Conclusions

In this study, we reviewed statistical methods that have important implications for estimating treatment effects. In conclusion, graphical displays capture different but essential aspects of treatment effects that should be described in the estimand framework. Researchers need to select appropriate methods, depending on the treatment and disease.

## Supplementary Information

**Additional file 1: Supplementary Fig. 1.** Graphs for simulated data by scenarios. Left: terminal trajectories of QOL; Right: survival curves.

**Additional file 2: Supplementary Method 1.** Simulation data generation.

**Additional file 3: Supplementary Method 2.** SAS program for simulation.

## Data Availability

Simulated data can be generated from the SAS code in the Supplementary Material (Supplementary Method 2).

## Abbreviations

MMRM: Mixed-effect models for repeated measures
OS: Overall survival
PRO: Patient-reported outcome
QOL: Quality of life
SACE: Survivor average causal effect
SISAQOL: Setting International Standards for the Analysis of Quality of Life
TTD: Time to deterioration
