# Supplementary material for: Statistical methods and graphical displays of quality of life with survival outcomes in oncology clinical trials for supporting the estimand framework
Source: BMC Med Res Methodol. 2022 Oct 4;22:259. doi: 10.1186/s12874-022-01735-1 (PMC9531431; doi:10.1186/s12874-022-01735-1)
Supplement: Supplementary file 1 — Additional file 1: Supplementary Fig. 1. Graphs for simulated data by scenarios. Left: terminal trajectories of QOL; Right: survival curves. [file 12874_2022_1735_MOESM1_ESM.pdf]

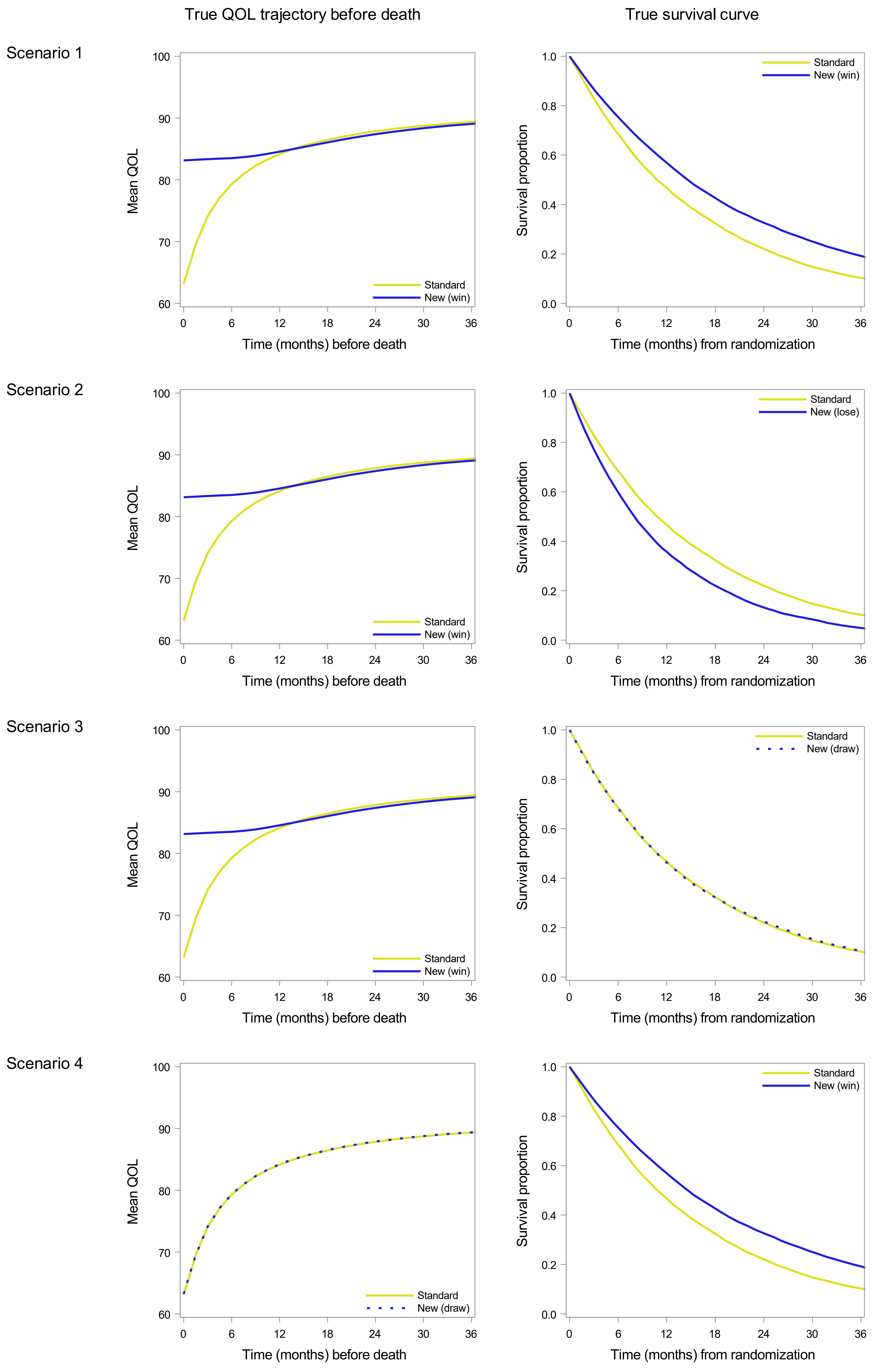

Supplementary Figure 1. Graphs for simulated data by scenarios
Left: terminal trajectories of QOL; Right: survival curves.
