# Supplementary material for: Statistical methods and graphical displays of quality of life with survival outcomes in oncology clinical trials for supporting the estimand framework
Source: BMC Med Res Methodol. 2022 Oct 4;22:259. doi: 10.1186/s12874-022-01735-1 (PMC9531431; doi:10.1186/s12874-022-01735-1)
Supplement: Supplementary file 2 — Additional file 2: Supplementary Method 1. Simulation data generation. [file 12874_2022_1735_MOESM2_ESM.docx]

**Supplementary Method 1: Simulation data generation**

For patients $i=1$ to $20,000$, treatment group $A_{i}$ was generated from a Bernoulli distribution with $p=0.5$, baseline QOL (${qol0}_{i}$) from the uniform distribution [$50-100$], and sex (${sex}_{i}$) from a Bernoulli distribution with $p=0.5$. The model for generating survival outcome was:

$$\lambda_{i}\left( t \right)=\lambda_{0}\left( t \right)\exp(\alpha_{A}A_{i}+\alpha_{1}{qol0}_{i}+\alpha_{2}{sex}_{i})$$

where $\lambda_{0}\left( t \right)=\exp\left( -2.5 \right)$ is the baseline hazard function, $\alpha_{1}=-0.005$, and $\alpha_{2}=0.2$. We varied $\alpha_{A}$ between scenarios. $\alpha_{A}=0.3$ and $-0.3$ correspond to hazard ratios of 0.74 and 1.35, respectively.

The model for generating longitudinal QOL from death was:

$$Y_{i}\left( t^{*} \right)=\beta\left( t^{*} \right)+\{\beta_{A}\left( t^{*} \right)+\beta_{adj}\left( t \right)\}A_{i}+\beta_{1}{qol0}_{i}+\beta_{2}{sex}_{i}+b_{i}+\varepsilon_{i}(t^{*})$$

where $t^{*}$ is the months from death, $b_{i}$ is the random intercept with the variance of 36, and the residual $\varepsilon_{i}\left( t^{*} \right)$ has the variance of 16. Here, $\beta\left( t^{*} \right)=140-\left\{ {30}/\left( 1+0.2t^{*} \right) \right\}$, $\beta_{1}=0.3$, and $\beta_{2}=1$. We varied $\beta_{A}\left( t^{*} \right)$ between scenarios. We made the average QOL at baseline equal between groups using the adjustment term $\beta_{adj}\left( t \right)$, which had different forms between scenarios, where $t$ is the months from randomization.

The parameters varying between scenarios were:

|  | $\beta_{A}\left( t^{*} \right)$ | $\beta_{adj}\left( t \right)$ | $\alpha_{A}$ |
| --- | --- | --- | --- |
| Scenario 1 | $30exp\left( -0.23t^{*}-0.92 \right)$ | $-4.7exp\left( -0.23t \right)$ | $-0.3$ |
| Scenario 2 | $30exp\left( -0.23t^{*}-0.92 \right)$ | $-3.7exp\left( -0.23t \right)$ | $0.3$ |
| Scenario 3 | $30exp\left( -0.23t^{*}-0.92 \right)$ | $-4exp\left( -0.23t \right)$ | $0$ |
| Scenario 4 | $0$ | $0$ | $-0.3$ |

Repeated measurements of QOL were assumed to be taken every 3 months until death. Censoring was assumed to be independent of the longitudinal QOL and survival and was generated by $G\times I\left( G<c \right)+c\times I(G\geq c)$, where $G$ follows a gamma distribution and $c$ is the upper bound of the censoring distribution. The shape and scale parameters of $G$ equal 1.55 and 14.3, respectively.
