# Supplementary material for: Statistical methods and graphical displays of quality of life with survival outcomes in oncology clinical trials for supporting the estimand framework
Source: BMC Med Res Methodol. 2022 Oct 4;22:259. doi: 10.1186/s12874-022-01735-1 (PMC9531431; doi:10.1186/s12874-022-01735-1)
Supplement: Supplementary file 3 — Additional file 3: Supplementary Method 2. SAS program for simulation. [file 12874_2022_1735_MOESM3_ESM.docx]

**Supplementary Method 2: SAS program for simulation**

**%macro** ***scenario***;

%GLOBAL n bm ba psi1 psi2 baseline alphaa alpha1 alpha2 shape scale ;

%do;

%let n = 20000; *sample size;

%let bm = 70 - (30/(1 + 0.2*t_star)) ; %let ba = &ba.; %let psi1 = 0.3; %let psi2 = 1; * QoL trajectory;

%let baseline = exp(-2.5); %let alphaa = &alphaa. ; %let alpha1 = -0.005; %let alpha2 = 0.2; * Hazard function ;

%let shape = 1.55; %let scale = 14.3 ; * Gamma dist for censoring ;

%end;

**%mend**;

%***scenario***;

**%macro** ***out***;

ODS EXCLUDE ALL;

proc format ; value arm **0**='Standard' **1**='New'; run;

data a;

call streaminit(**1**);

do parameter =**1** to **1**;

do repeat = **1** to **1**;

do pid=**1** to &n.;

if mod(pid,**2**)=**1** then Arm=rand('bernoulli',**0.5**);

if mod(pid,**2**)=**0** and lag(Arm)=**1** then Arm=**0**;

if mod(pid,**2**)=**0** and lag(Arm)=**0** then Arm=**1**;

qol0 = rand('Uniform', **50**, **100**);

sex = rand('Bernoulli', **0.5**);

b = rand('Normal', **0**, **6**);

Death1 = - ( log(rand('Uniform', **0**, **1**)) )/ (exp(**1***&alphaa. + &alpha1. * qol0 + &alpha2. * sex) * &baseline.) ;

Death0 = - ( log(rand('Uniform', **0**, **1**)) )/ (exp(**0***&alphaa. + &alpha1. * qol0 + &alpha2. * sex) * &baseline.) ;

if Arm = **1** then Death = Death1; else if Arm = **0** then Death = Death0;

G = rand('Gamma', &shape. ) * &scale. ;

Censor = G * (G < **36**) + **36** * (G >= **36**);

OS_Event = (Death <= Censor);

time = Death * (Death < Censor) + Censor * (Death >= Censor);

do t_star = **0** to **300** by **1**;

t_continum = Death - t_star;

t = ceil(Death - t_star);

Y1 = &bm. + **1*** &ba. + &psi1.*qol0 + &psi2.*sex + b + rand('Normal', **0**, **4**);

Y0 = &bm. + **0*** &ba. + &psi1.*qol0 + &psi2.*sex + b + rand('Normal', **0**, **4**);

if Arm = **1** then Y = Y1; else if Arm = **0** then Y = Y0;

output; end;

end;

end;

end;

format arm arm.;

run;

data a0;

set a;

where t=**0**;

keep pid y;

rename y=base;

run;

data a1;

merge a a0;

by pid;

Chg = Y - base;

if Chg < -**10** and Chg ne **.** then deterioration = **1**; else deterioration = **0**;

if mod(t, **3**) ne **0** then delete; *because QOL is measured every 3 months;

run;

proc sort data=a1(where=(t>=**0**)) out=a1;

by pid t;

run;

proc sort data=a1(where=(time >= t >=**0**)) out=obs1(drop= b death death1 death0 y1 y0 g censor t_continum);

by pid t;

run;

data obs2(drop = total t_star );

set obs1;

by pid;

retain total;

if first.pid then total = **0**;

total + deterioration;

if (total > **0**) then qol_event = **1**; else qol_event = **0**;

if qol_event = **1** then TTD_Event = **1**;

else if os_event = **1** and last.pid then TTD_Event = **2**;

else TTD_Event = **0**;

if qol_event =**1** then TTD_time = t; else TTD_time = time;

if last.pid OR TTD_event = **1**;

run;

data obs3;

set obs2;

by pid;

if first.pid ;

run;

* Mixed-effects model ;

proc mixed data=obs1;

class t arm pid;

model y = arm t arm*t qol0 sex;

repeated pid / type = ar(**1**);

lsmeans arm*t / pdiff cl;

ods output LSMeans=_lsm1 Diffs=_diff1(where=(t=_t));

run;

* Time to deterioration analysis;

proc lifetest data=obs3 plots=survival(atrisk= **0** to **36** by **6**);

time TTD_Time * TTD_Event(**0**) ;

strata Arm ;

ods output SurvivalPlot=SurvivalPlotData_TTD;

run;

* Survivor Average Causal Effect ;

data a1_sace;

set a1;

**%macro** sace(t); if death1>=&t. and death0>=&t. and t=&t. then do; sace = y1 - y0; sace_y1 = y1; sace_y0=y0; t_sace = &t.; end; **%mend**;

%***sace***(**3**)%***sace***(**6**)%***sace***(**9**)%***sace***(**12**)%***sace***(**15**)%***sace***(**18**)%***sace***(**21**)%***sace***(**24**)%***sace***(**27**)%***sace***(**30**)%***sace***(**33**)%***sace***(**36**)

run;

proc means data=a1_sace n mean stddev lclm uclm probt t;

var sace sace_y1 sace_y0;

class t_sace;

ods output Summary = sace;

run;

* Competing risk analysis;

proc phreg data= obs3;

class Arm(ref="Standard");

model TTD_Time * TTD_Event(**0**) = / RiskLimits EVENTCODE = **1**;

strata Arm;

baseline out = CIF cif = _all_ ;

run;

* Prioritized Outcomes ;

data obs4;

set obs1;

by pid;

if first.pid OR t=**12**;

run;

%let delta= 0;

data obs5;

set obs4;

by pid;

if last.pid;

if time<**12** then do; co=time; co_event=os_event; end;

if time>=**12** then do; co=y + &delta.; co_event=**1**; end;

run;

proc lifetest data=obs5 plots=survival;

time co * co_Event(**0**) ;

strata Arm ;

ods output ProductLimitEstimates= ProductLimitEstimates_co;

run;

data ProductLimitEstimates_co2;

set ProductLimitEstimates_co;

if co>=**12** then do;

co2=co; co=**.**;

end;

run;

**%mend**;

**%macro** ***figure***;

ODS SEELCT ALL;

ods region;

ods graphics / height=**3**in width=**3**in noborder;

proc sgplot data=_lsm1 noautolegend ;

styleattrs datacontrastcolors=(strongblue strongyellow );

series x = t y = estimate / group = arm lineattrs = (thickness = **2**) groupdisplay=cluster clusterwidth= **0.2** name="series";

scatter x = t y = estimate / group = arm markerattrs=(size=**5** symbol=circlefilled) groupdisplay=cluster clusterwidth= **0.2**;

keylegend "series" / location=inside position=topright noborder across=**1** valueattrs=(family=Arial size=**10**);

yaxis label="Means" values=(**60** to **100** by **10**) labelattrs=(family=Arial size=**10**) valueattrs=(family=Arial size=**10**);

xaxis label="Time (months) from randomization" values= (**0** to **36** by **6**) labelattrs=(family=Arial size=**10**) valueattrs=(family=Arial size=**10**);

run;

ods region ;

ods graphics / height=**3**in width=**3**in noborder;

proc sgplot data=SurvivalPlotData_TTD noautolegend ;

styleattrs datacontrastcolors=( strongblue strongyellow);

step x=time y=survival / group=stratum name='step' lineattrs=(thickness=**2**);

keylegend "step" / location=inside position=topright noborder across=**1** valueattrs=(family=Arial size=**10**);

yaxis values=(**0** to **1** by **0.2**) label="1 - Probability of deterioration" labelattrs=(family=Arial size=**10**) valueattrs=(family=Arial size=**10**);

xaxis values=(**0** to **36** by **6**) label="Time (months) from randomization" labelattrs=(family=Arial size=**10**) valueattrs=(family=Arial size=**10**);

run;

data _last;

set ProductLimitEstimates_co2;

where co ne **.** and survival ne **.**;

by stratum co;

if last.stratum ne **1** then delete;

co=**.**; co2=**50**;

run;

data ProductLimitEstimates_co2; set ProductLimitEstimates_co2 _last; if **0**<co2<**50** then co2=**50**; run;

proc sort data=ProductLimitEstimates_co2;

by stratum co2 co;

run;

proc format ; value arm **2**='Standard' **1**='New'; run;

ods region;

ods graphics / height=**3**in width=**3**in noborder;

proc sgplot data=ProductLimitEstimates_co2 noautolegend ;

styleattrs datacontrastcolors=( strongblue strongyellow);

step x=co y=failure / group=stratum name='step' lineattrs=(thickness=**2** pattern=**1**);

step x=co2 y=failure / group=stratum lineattrs=(thickness=**2** pattern=**1**) x2axis;

keylegend 'step' / location=inside position=bottomright noborder across=**1** valueattrs=(family=Arial size=**10**);

yaxis values=(**0** to **1** by **0.2**) label="Cumulative Probability" labelattrs=(family=Arial size=**10**) valueattrs=(family=Arial size=**10**);

xaxis values=(**0** to **12** by **6**) label="Time to death (month)" offsetmax=**0.5** labelpos=datacenter labelattrs=(family=Arial size=**10**) valueattrs=(family=Arial size=**10**);

x2axis values=(**50** to **110** by **10**) label="QoL at 12 mo" offsetmin=**0.5** labelpos=datacenter labelattrs=(family=Arial size=**10**) valueattrs=(family=Arial size=**10**);

refline **12** / axis=x;

format stratum arm.;

run;

ods region;

ods graphics / height=**3**in width=**3**in noborder;

proc sgplot data=CIF noautolegend;

styleattrs datacontrastcolors=( strongblue strongyellow);

step x=TTD_time y=CIF / group=Arm name='step' lineattrs=(thickness=**2**);

yaxis values=(**0** to **1** by **0.2**);

xaxis values=(**0** to **36** by **6**);

keylegend "step" / location=inside position=topright noborder across=**1** valueattrs=(family=Arial size=**10**);

yaxis label="Probablity of deterioration" values=(**0** to **1** by **0.2**) labelattrs=(family=Arial size=**10**) valueattrs=(family=Arial size=**10**);

xaxis label="Time (months) from randomization" values= (**0** to **36** by **6**) labelattrs=(family=Arial size=**10**) valueattrs=(family=Arial size=**10**) ;

run;

**%mend**;

options leftmargin=**.25**in rightmargin=**.25**in topmargin=**.25**in bottommargin=**.25**in nonumber nodate papersize=(**18**in **18**in);

ods pdf file="Figure2.pdf" notoc startpage=no ;

title ;

ods layout gridded rows=**5** columns=**4** column_widths=(**3.5**in **3.5**in **3.5**in **3.5**in);

ods escapechar='^';

ods region;

ods pdf text="^S={JUST=C fontsize=11pt font_face=Arial} MMRM analysis";

ods region;

ods pdf text="^S={JUST=C fontsize=11pt font_face=Arial} TTD analysis";

ods region;

ods pdf text="^S={JUST=C fontsize=11pt font_face=Arial} Prioritized Composite Outcome analysis";

ods region;

ods pdf text="^S={JUST=C fontsize=11pt font_face=Arial} Semi-competing risk analysis";

*Scenario 1;

%let ba = (50*exp(-0.23*t_star - 0.92) - 4.7*exp(-abs(0.23*t)));

%let alphaa = -0.3;

%***out***;

%***figure***;

*Scenario 2;

%let ba = (50*exp(-0.23*t_star - 0.92) - 3.7*exp(-abs(0.23*t)));

%let alphaa = -0.3;

%***out***;

%***figure***;

*Scenario 3;

%let ba = (50*exp(-0.23*t_star - 0.92) - 4*exp(-abs(0.23*t)));

%let alphaa = 0;

%***out***;

%***figure***;

*Scenario 4;

%let ba = 0;

%let alphaa = -0.3;

%***out***;

%***figure***;

ods layout end;

ods pdf close;
